# Research progress on the mechanisms by which gut microbiota dysbiosis remodels the bone marrow neuroimmune microenvironment to drive age-related osteoporosis

**DOI:** 10.3389/fimmu.2026.1884778

**Published:** 2026-09-29

**Authors:** Haoze Zhang, Zhong Wang, Tianwen Miao, Xingwen Xie, Ning Li

**Affiliations:** 1 University of Gansu Traditional Chinese Medicine, Lanzhou, China; 2 Affiliated Hospital of Gansu University of Traditional Chinese Medicine, Lanzhou, China

**Keywords:** age-related osteoporosis, bone marrow microenvironment, bone remodeling, gut microbiota dysbiosis, inflammaging, neuroimmune axis, osteoimmune imbalance

## Abstract

Age-related osteoporosis arises from the interplay between impaired bone remodeling, immune remodeling, and chronic low-grade inflammation during aging. Beyond local degeneration in bone tissue, alterations in the gut microbiota, mucosal barrier, and autonomic nervous regulation may reshape the bone marrow immune environment and amplify osteoclastogenic responses. This review integrates evidence linking the gut, neural regulation, and the bone marrow immune microenvironment. It summarizes their potential contributions to age-related bone loss and provides a conceptual framework for understanding disease mechanisms, improving risk stratification, and optimizing intervention strategies.

## 1 Introduction

Osteoporosis is the most frequent skeletal condition in midlife and old age, with osteoporotic fractures as the ultimate adverse outcome of osteoporosis. Osteoporotic fractures are associated with functional impairment, dependence, and higher mortality (1). The International Osteoporosis Foundation reports that osteoporotic fractures affect approximately 30% of women and 20% of men over the age of 50 worldwide (2). These data indicate that osteoporosis is not only a disease affecting women, but also an important aging-related skeletal disorder in both sexes. A nationwide population-based cross-sectional study in China reported that the prevalence of osteoporosis among adults aged 40 years and older was 20.6% in women and 5.0% in men (3). The same study also found vertebral fractures in 10.5% of men and 9.7% of women. The China Community-based Cohort of Osteoporosis showed that the prevalence of osteoporosis in middle-aged and older adults was associated with age, sex, BMI, educational level, smoking status, prior fracture history, and geographic region, with marked regional differences (4). Evidence from Wuhan, Beijing, and the Uyghur population in Xinjiang indicated that low bone mass or osteoporosis was associated with alterations in gut microbiota composition. Notably, a community-based study of postmenopausal osteoporosis in Beijing used integrated metagenomic and fecal metabolomic analyses. The results showed that changes in gut microbiota and microbial metabolites were significantly correlated with bone mineral density (BMD) and bone turnover markers (5–8).

Age-related osteoporosis has traditionally been attributed to impaired bone formation, excessive bone resorption, senescence of bone marrow mesenchymal stem/stromal cells (BMSCs), and deterioration of the bone marrow microenvironment. However, these local alterations do not fully explain the progressive, systemic, and heterogeneous nature of age-related bone loss. The bone marrow is not only a site of bone remodeling but also a critical niche for hematopoietic stem cell maintenance and for the generation, differentiation, and functional regulation of immune cells (9).

Aging is accompanied by profound remodeling of the immune system. T cells, B cells, macrophages, and dendritic cells undergo age-related changes in phenotype and function. Together, immunosenescence and inflammaging disrupt the RANKL/OPG axis, increase exposure to pro-inflammatory cytokines, and enhance osteoclastogenic signaling. Consequently, bone homeostasis gradually shifts from protective immunoregulation toward a resorption-dominant state (10).

Gut microbiota dysbiosis has recently emerged as an important upstream regulator of bone homeostasis. During aging, alterations in microbial composition and metabolism, together with impaired intestinal barrier integrity, may promote the systemic dissemination of gut-derived inflammatory signals (11, 12). These signals may reach the bone marrow and contribute to remodeling of its immune microenvironment (12, 13). In addition, gut microbiota and their metabolites may influence autonomic nervous system activity through vagal and sympathetic pathways. Through these routes, intestinal signals may modulate the activation state of bone marrow immune cells and the immune networks that regulate bone remodeling (14, 15). Although gut microbiota dysbiosis, inflammaging, and neural regulation have each been implicated in disordered bone metabolism, their interactions in age-related osteoporosis remain incompletely understood (16–18). In particular, the mechanistic links among gut microbiota dysbiosis, neuroimmune dysregulation, remodeling of the bone marrow immune microenvironment, and impaired bone remodeling have not been systematically clarified.

Therefore, this review summarizes the pathological basis of age-related osteoporosis, focusing on how aging-associated gut microbiota dysbiosis, intestinal barrier dysfunction, and gut-derived inflammatory signals reshape the bone marrow immune microenvironment. We also discuss the potential roles of impaired vagal anti-inflammatory signaling and abnormal sympathetic outflow in promoting osteoimmune imbalance within the bone marrow. By positioning the bone marrow neuroimmune microenvironment as a key intersection between gut-derived signals, neural regulation, and osteoimmune remodeling, this review provides an immunological framework for understanding disease mechanisms and for developing risk-stratification strategies and precision interventions in age-related osteoporosis.

## 2 Pathological basis of age-related osteoporosis

Age-related osteoporosis results from the combined aging-related deterioration of bone tissue, the bone marrow microenvironment, and the immune regulatory network (19). With advancing age, bone remodeling gradually shifts from a dynamic balance between bone formation and bone resorption toward a sustained negative remodeling balance, leading to bone loss, deterioration of trabecular microarchitecture, and reduced bone strength (20). Unlike postmenopausal osteoporosis, in which estrogen deficiency is a major pathological driver, age-related osteoporosis reflects the cumulative effects of impaired osteogenic capacity, bone marrow niche degeneration, chronic low-grade inflammation, and osteoimmune dysregulation (21). Therefore, its pathogenesis should not be attributed solely to changes in osteoblasts and osteoclasts. Increasing attention should also be directed toward the local regulatory microenvironment formed by the bone marrow stroma, blood vessels, nerves, and immune cells. **Figure 1** schematically illustrates aging-driven alterations in bone remodeling and the bone marrow microenvironment.

**Figure 1:**
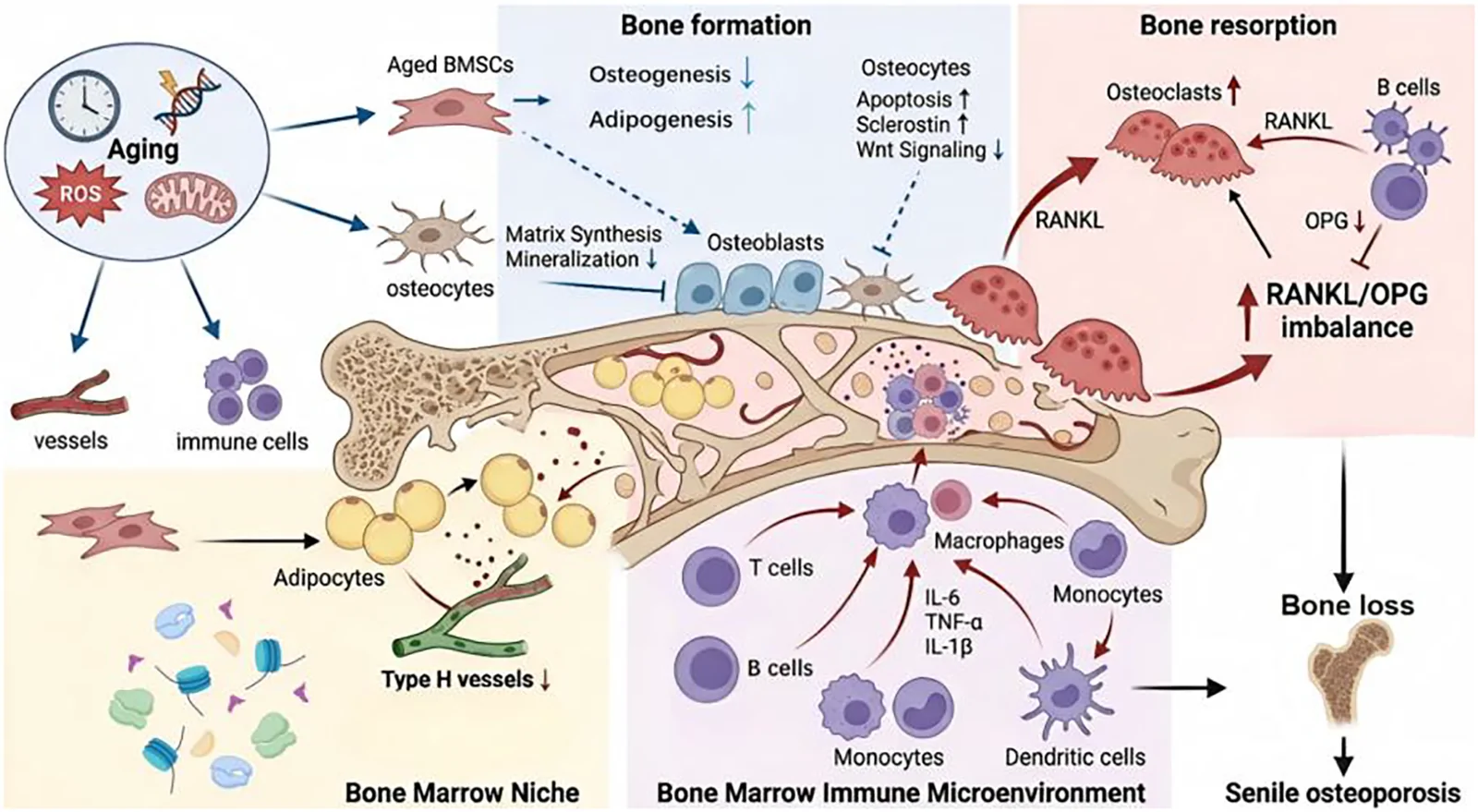
Aging-related alterations in bone remodeling and the bone marrow microenvironment. Aging impairs BMSC osteogenic differentiation, promotes marrow adiposity, reduces osteoblast activity, and enhances RANKL-mediated osteoclast activation. Together with vascular degeneration and immune-cell activation, these changes shift bone homeostasis toward a pro-inflammatory and pro-osteoclastogenic state, leading to age-related bone loss.

### 2.1 Aging shifts bone remodeling toward a negative balance

Bone tissue undergoes continuous remodeling throughout life. The preservation of bone mass relies on the coordinated coupling of osteoblast-mediated bone formation with osteoclast-mediated bone resorption. During aging, this coupling is progressively disrupted because of impaired osteogenic capacity and relatively increased osteoclast-mediated bone resorption, resulting in bone loss and deterioration of bone microarchitecture (17, 18). On the bone-forming side, aging compromises the self-renewal and osteogenic differentiation potential of BMSCs, thereby reducing the availability of mature osteoblasts and impairing matrix synthesis and mineralization (19). In parallel, deterioration of the osteocyte network, increased osteocyte apoptosis, and elevated sclerostin expression suppress Wnt/β-catenin signaling and further limit osteoblast activity (20, 21). In addition to impaired bone formation, bone resorption becomes relatively enhanced with aging. In aged bone, increased osteoclast numbers, elevated RANKL expression by senescent osteocytes, and insufficient OPG-mediated inhibition collectively shift the RANKL/OPG axis toward a pro-osteoclastogenic state. These changes promote the formation and activation of osteoclasts, thereby keeping a continuous negative remodeling balance (22, 23).

### 2.2 Age-related deterioration of the bone marrow microenvironment

The bone marrow microenvironment serves as an important regulatory system that controls bone homeostasis. It consists of BMSCs, hematopoietic cells, endothelial cells, immune cells, ECM, and soluble factors (22). Given that BMSCs serve as the main progenitors of osteoblastic cells, they are considered a crucial factor in the development of bones. In the context of aging, BMSCs face cell-cycle arrest, oxidative stress, mitochondrial dysfunction, and diminished osteogenic capacity. Their lineage commitment also shifts from osteogenesis toward adipogenesis, characterized by suppression of osteogenic programs such as Runx2 and Osterix and upregulation of adipogenic transcription factors such as PPARγ and C/EBPα (23–26). This lineage shift is also influenced by epigenetic remodeling. Age-related changes in DNA modifications and the accumulation of repressive histone marks may disrupt the transcriptional balance that governs osteogenic and adipogenic lineage commitment in BMSCs. Studies have shown that increasing donor age is associated with 5hmC changes at specific CpG sites in BMSCs, together with 5mC loss at some loci. These findings suggest that remodeling of DNA methylation and hydroxymethylation may represent an important epigenetic feature of age-related functional changes in BMSCs (27). In addition, the proportions of H3K27me3- and H3K9me3-positive MSCs are increased in the bone marrow of aged mice. Functional studies further showed that KDM6B and KDM4B promote MSC osteogenic differentiation by removing H3K27me3 and H3K9me3, respectively. Inhibition of these two demethylases impairs osteogenic potential and enhances adipogenic differentiation (28). Expansion of bone marrow adiposity not only replaces osteogenic marrow components but also impairs bone formation and promotes bone resorption through inflammatory and paracrine mechanisms, thereby further aggravating bone loss (29, 30). Age-related deterioration of the local marrow niche also contributes to impaired bone homeostasis. Reduced microvascular density, insufficient perfusion, loss of type H vessels, and endothelial dysfunction weaken the nutritional, recruitment, and reparative support required by BMSCs and osteoprogenitor cells (31–33). In addition, alterations in marrow niche architecture and extracellular matrix signaling may impair BMSC anchorage and osteogenic differentiation (34–36). Age-associated changes in the bone marrow microenvironment may also affect neural inputs and immune-cell composition. Consequently, the marrow niche gradually shifts from a homeostatic environment that supports osteogenesis and tissue repair toward a pro-inflammatory and pro-osteoclastogenic niche that favors bone loss (10, 37, 38).

### 2.3 Osteoimmune imbalance

Bone marrow immune cells serve as key mediators at the interface of inflammation, neural regulation, and bone remodeling (39–41). T cells, B cells, macrophages, monocytes, and dendritic cells are all involved in osteoimmune regulation (42). Among adaptive immune cells, T cells represent an important source of signals that promote osteoclastogenesis. Fessler et al. reported that senescence-like CD4+CD28− T cells showed higher RANKL expression than CD28+ T cells, and that IL-15 stimulation further enhanced RANKL expression in these cells (43). After RANKL binds to RANK on the surface of osteoclast precursors, it activates NF-κB and MAPK signaling through TRAF6. This process induces the expression of AP-1 components, including c-Fos and c-Jun, as well as NFATc1. NFATc1 then drives the transcription of osteoclast functional genes, such as TRAP and CTSK, thereby promoting osteoclast formation and bone resorption (44). By contrast, B cells appear to exert a relatively protective effect on bone homeostasis. In the SAMP6 aging model, the number of bone marrow pro-B cells is reduced and is accompanied by increased oxidative stress. This activates Fos- and Jun-mediated AP-1 signaling and upregulates CCL3 expression. Elevated CCL3 acts through its receptor CCR1 on monocytes, macrophages, and osteoclast precursors, thereby enhancing osteoclast differentiation. In contrast, CCR1 blockade can partially reverse this pro-osteoclastogenic effect (45). Innate immune cells also contribute substantially to osteoimmune dysregulation during aging. The monocyte–macrophage lineage is not only an important source of inflammatory cytokines such as IL-6, TNF-α, and IL-1β, but also gives rise to osteoclast precursors (46). Aging reshapes the bone marrow macrophage/osteoclast lineage and enhances osteoclast differentiation. In this context, Cebpd has been recognized as a key regulator of age-associated osteoclastogenesis and bone resorption (47). Aging-associated neutrophils regulate bone metabolism via paracrine signaling. During aging, TGFβ1+CCR5+ neutrophils accumulate within the bone marrow and release TGFβ1, thereby inducing TRAF3 degradation in mesenchymal progenitor cells and activating the NF-κB/CCL5 signaling axis. Together, these events promote osteoclastogenesis, suppress bone formation, and ultimately contribute to age-related bone loss and osteoporosis in mice (48). Moreover, bone marrow immune cells may serve as downstream effectors through which neural signals regulate bone remodeling, as neural inputs can modify immune-cell activation states and cytokine profiles (49). Thus, bone marrow immune cells integrate inflammatory and neural signals. Disturbance of the RANKL/OPG balance, together with dysregulated inflammatory cytokine production, ultimately drives imbalanced bone remodeling during aging.

## 3 Aging-associated gut microbiota dysbiosis

The gut microbiota serves as a key regulator of host nutrient metabolism, intestinal barrier integrity, and immune homeostasis. During aging, gradual alterations in microbial composition, metabolic activity, and mucosal barrier homeostasis may shift the gut from a relatively stable symbiotic state toward low-grade inflammation and functional disturbance (50). In the context of bone metabolism, the effects of the gut microbiota extend beyond the gastrointestinal tract. The gut microbiota may influence distant organs through microbial metabolites, increased intestinal permeability, and the systemic dissemination of immunostimulatory signals (51). In older individuals, dysbiosis and intestinal barrier impairment may reinforce each other, facilitating the progression from local intestinal disturbance to systemic inflammation and metabolic dysfunction. These age-related alterations may provide persistent upstream inputs that contribute to reconfiguration of the bone marrow immune microenvironment (12, 14). **Figure 2** schematically illustrates aging-associated gut microbiota dysbiosis, intestinal barrier dysfunction, and inflammatory signaling to the bone marrow.

**Figure 2:**
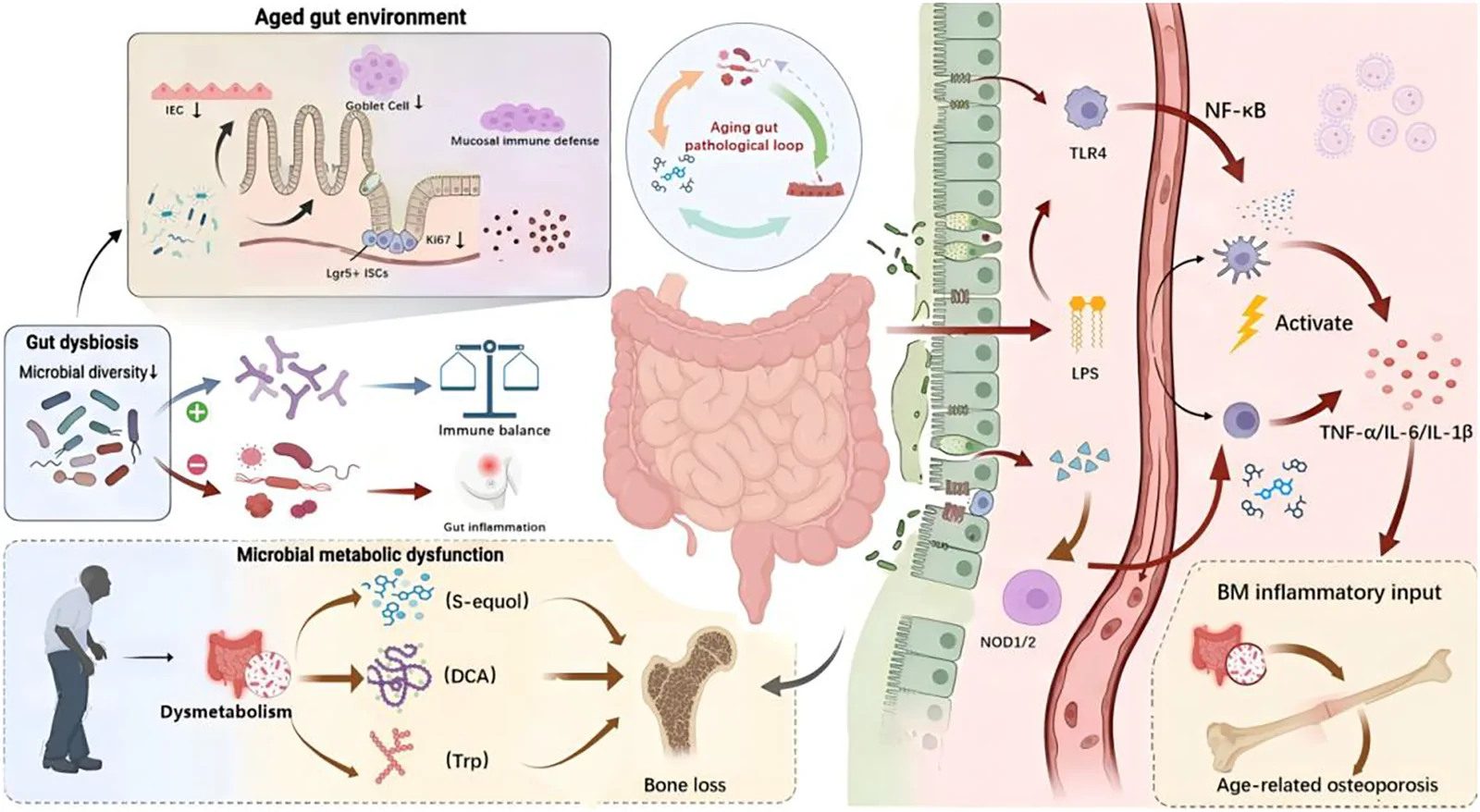
Gut-derived inflammatory and metabolic inputs to the aging bone marrow. Aging-associated dysbiosis and intestinal barrier impairment facilitate the translocation of microbial products, including LPS and peptidoglycan, which activate inflammatory pathways such as TLR4/NF-κB and NOD1/2. Altered microbial metabolism further contributes to systemic inflammation, bone marrow immune remodeling, and age-related bone loss.

### 3.1 Aging alters the composition and function of the gut microbiota

Aging induces both compositional and functional changes in the gut microbiota. In older individuals, the gut microbiota is often characterized by reduced diversity, depletion of beneficial commensals, and relative enrichment of opportunistic pathogens and pro-inflammatory taxa. As a result, the stable symbiotic state of the intestinal ecosystem is disrupted, and the microbial community gradually shifts from a homeostatic profile toward a pro-inflammatory one (52, 53). Among beneficial bacteria, Lactobacillus, Bifidobacterium, and Akkermansia are closely associated with bone metabolic homeostasis. Lactobacillus may exert bone-protective effects by regulating the Th17/Treg balance, reducing IL-17 and TNF-α levels, and downregulating RANKL expression (54). Bifidobacterium can enhance Breg–Treg-mediated anti-inflammatory responses, decrease pro-inflammatory cytokines such as IL-6, IL-17, and TNF-α, and inhibit RANKL-induced osteoclast differentiation (55). Akkermansia may also contribute to bone homeostasis by promoting Foxp3^+^ Treg responses and suppressing IL-6-, TNF-α-, and RANKL-related signaling (56). During aging, depletion of these beneficial taxa may weaken the protective regulation of intestinal immunity, inflammation, and bone metabolism. In contrast, several opportunistic pathogens and pro-inflammatory taxa are relatively enriched in individuals with osteoporosis, including Clostridium sensu stricto, Bacteroides, and Intestinibacter (57). These taxa may represent a pro-inflammatory dysbiotic intestinal signature of age-related osteoporosis and may be associated with immune dysregulation, IL-17–related inflammatory responses, and increased osteoclastogenic activity (58).

Beyond changes in microbial composition, aging also affects the metabolic functions of the gut microbiota. Individuals with osteoporosis may show reduced serum S-equol levels and altered fecal metabolic profiles, mainly involving amino acid metabolism and tryptophan-related pathways (59). Metabolomic studies have shown that osteoporosis-associated metabolic abnormalities are concentrated in the biosynthetic pathways of phenylalanine, tyrosine, and tryptophan, and may be influenced by sex differences (60). Aging can also alter bile acid metabolic profiles (61). Animal studies have shown age-related alterations in secondary bile acid levels in aged mice. In particular, reduced hyodeoxycholic acid levels occur in parallel with bone loss. After transplantation of a young gut microbiota, hyodeoxycholic acid levels increase, bone resorption is suppressed, and bone loss is partially alleviated (62).

#### 3.1.1 Bone-protective effects of short-chain fatty acids

Short-chain fatty acids (SCFAs) are major metabolites produced by the gut microbiota through the fermentation of dietary fiber. Acetate, propionate, and butyrate are the most representative SCFAs. Beyond serving as energy substrates, SCFAs can transmit gut microbial signals to immune cells, osteoblast-lineage cells, and osteoclast precursors. These effects are mediated through membrane receptors such as GPR41, GPR43, and GPR109A, as well as through HDAC inhibition. Through these mechanisms, SCFAs contribute to the regulation of bone remodeling homeostasis (63). At the immune level, butyrate and propionate promote the differentiation of naive CD4^+^ T cells into peripheral induced Treg cells through HDAC inhibition. Butyrate increases histone acetylation at the Foxp3 promoter and at the CNS1, CNS2, and CNS3 regions. This enhances Foxp3 expression and promotes Treg generation (64). A Treg-dominant immune state may further restrain Th17/IL-17-related inflammatory responses. In osteoporosis-related models, increasing gut microbiota-derived SCFAs improves Th17/Treg imbalance, reduces inflammation, and attenuates bone loss (65, 66). This immunomodulatory effect may also amplify osteogenic signaling. The Wnt/β-catenin pathway is a key axis for osteoblast differentiation and bone matrix formation. Studies have shown that supplementation with LGG or butyrate expands Treg cells in the gut and bone marrow. It also induces bone marrow CD8^+^ T cells to produce Wnt10b, thereby activating osteogenic Wnt signaling and promoting bone formation (67). In the context of PTH-induced bone anabolism, microbiota-derived butyrate may also contribute to bone formation through the GPR43, Treg, CD8^+^ T cell, and Wnt10b axis (68). During bone resorption, SCFAs can directly reduce the responsiveness of osteoclast precursors to RANKL-induced differentiation. In M-CSF- and RANKL-induced osteoclastogenic systems, propionate and butyrate reduce the formation of TRAP^+^ multinucleated osteoclasts and inhibit bone resorption activity. This effect is linked to metabolic reprogramming during early differentiation. Specifically, SCFAs shift osteoclast precursors away from oxidative phosphorylation toward a glycolytic energy-stress state, leading to the downregulation of key osteoclastogenic signals such as TRAF6 and NFATc1 (65).

#### 3.1.2 Neuroimmune regulatory effects of tryptophan metabolites

Tryptophan is an aromatic amino acid that links the gut microbiota, barrier function, and immune homeostasis. Different branches of tryptophan metabolism may exert distinct effects on bone remodeling. During aging-related gut dysbiosis, tryptophan metabolism appears to shift from reduced microbial indole production toward enhanced activation of the host kynurenine pathway (69). Experimental studies have shown that microbiota-derived tryptophan metabolites, including indole-3-acetic acid and indole-3-propionic acid, can repair intestinal barrier function through AhR-dependent mechanisms. This effect may alleviate ovariectomy-induced bone loss (70). In contrast, elevated kynurenine in the context of aging and chronic inflammation is more commonly associated with adverse changes in bone metabolism. Clinical evidence shows that higher bone marrow kynurenine levels in individuals aged 65 years and older are associated with an increased likelihood of fragility hip fracture, lower total femur BMD, and higher bone marrow levels of TRAP-5b and RANKL (71). As circulating metabolic signals, tryptophan metabolites may also link the gut, neuroimmune regulation, and the bone marrow immune microenvironment. Kynurenine can cross the blood-brain barrier via LAT1 and enter the brain. Its downstream metabolites may affect the central neuroimmune state through NMDA receptor activation, oxidative stress, and glial inflammatory responses (72, 73). Microbiota-derived indole-3-propionic acid can also enter brain tissue and regulate microglial activation and neuroinflammation through AhR/NF-κB-related signaling (74). In the bone marrow microenvironment, elevated kynurenine can act as an AhR ligand and enhance the responsiveness of osteoclast precursors to RANKL-induced differentiation. It upregulates c-Fos and NFATc1 and increases the formation of TRAP^+^ multinucleated osteoclasts, thereby promoting a shift toward enhanced bone resorption (75).

#### 3.1.3 Sexual dimorphism of the gut microbiota

The gut microbiota exhibits pronounced sex-related differences, which may be particularly relevant to age-related osteoporosis. A metagenomic analysis of 2,338 adults, validated in independent cohorts from China, Israel, and the Netherlands, showed that premenopausal women had higher gut microbial diversity than men, as well as higher abundances of several species associated with metabolic health. These sex differences gradually diminished with age, suggesting that sex hormones may help shape the gut microbiota (76). The longitudinal SWAN study further showed that, as 65 women transitioned from premenopause to postmenopause, biomarkers related to intestinal epithelial injury and microbial translocation increased. FABP2, LBP, and sCD14 increased by 22.8%, 3.7%, and 8.9%, respectively. Higher levels of intestinal permeability markers were also associated with increased inflammation and lower bone mineral density at the lumbar spine and total hip (77).

Findings from human intervention studies remain inconsistent. In 90 older women with low bone mass, Lactobacillus reuteri reduced the loss of tibial volumetric bone mineral density by approximately half over 12 months (78). However, in 239 early postmenopausal women, a 2-year intervention did not demonstrate a clear bone-protective effect (79). Taken together, these findings suggest that sex, menopausal status, age, and baseline bone mass may jointly shape the relationship between the gut microbiota and bone metabolism. In gut–bone axis research, these factors should not be treated merely as covariates, but rather as key stratification factors in study design and analysis.

### 3.2 Impairment of the intestinal barrier

The intestinal barrier is composed of intestinal epithelial cells, intercellular tight junctions, the mucus layer, and local immune components (80). An intact intestinal barrier restricts the passage of luminal bacteria and their products across the epithelium, thereby maintaining local homeostasis. With aging, the intestinal epithelial barrier becomes progressively impaired. The expression of tight junction proteins, such as occludin, claudins, and ZO-1, is reduced. Gut microbiota linked to age-related osteoporosis may further aggravate this barrier dysfunction and promote bone loss (81). Concurrently, aging is accompanied by a reduction in the number of colonic goblet cells and mucin content, which may compromise mucus barrier function (82). Studies have also reported disorganized intestinal villi, abnormal crypt morphology, and disrupted epithelial continuity during aging. These findings suggest that age-related intestinal barrier damage involves broad degeneration, ranging from tissue architecture to secretory function (83, 84). In addition, epithelial proliferation and renewal are impaired, as indicated by reduced activity of Lgr5-positive intestinal stem cells and decreased Ki67 expression (85). Impaired intestinal epithelial autophagy may also contribute to barrier dysfunction. Autophagy removes damaged mitochondria, misfolded proteins, and inflammatory stress products. In this way, it limits ROS accumulation, endoplasmic reticulum stress, and cell death. It also helps maintain the turnover homeostasis of tight junction proteins, including claudins, occludin, and ZO-1. Age-related impairment of autophagic flux may therefore disrupt tight junction organization, increase barrier permeability, and amplify low-grade inflammation (86, 87).

Local immune defense is also weakened, as reflected by reduced secretory immunoglobulin A (SIgA) and an imbalanced antimicrobial peptide profile (88, 89). M cells are specialized epithelial cells in the follicle-associated epithelium of Peyer’s patches. They are responsible for luminal antigen sampling and transepithelial transport. Aging may downregulate maturation- and chemotaxis-related molecules, such as Spi-B, GP2, and CCL20. This reduces the number of mature M cells and impairs the delivery of particulate antigens to dendritic cells and B cells in the subepithelial dome. As a result, germinal center reactions in Peyer’s patches, IgA plasma cell generation, and SIgA-mediated mucosal immune surveillance may be compromised (90, 91). Paneth cells are a major source of antimicrobial peptides in small intestinal crypts. They secrete molecules such as α-defensins and lysozyme, which help limit bacterial colonization in the crypt region and maintain intestinal homeostasis. Aging may reduce α-defensins and lysozyme, while increasing antimicrobial-related molecules such as Reg3b, Reg3g, β-defensin 1, and angiogenin 4. These changes suggest a stress-related imbalance in the antimicrobial peptide profile of the aged intestine (92).

Once the intestinal barrier is compromised, microbial components and metabolites in the gut lumen can more readily cross the epithelium and gain access to the lamina propria and systemic circulation. Lipopolysaccharide (LPS), for example, can be detected by Toll-like receptor 4 (TLR4) expressed on macrophages. This activates NF-κB signaling via a MyD88-dependent pathway and induces production of the inflammatory cytokine IL-6 (93). Peptidoglycan (PGN)-derived muropeptides can access the cytosol and be sensed by cytosolic NOD1 and NOD2 receptors. NOD2 primarily recognizes muramyl dipeptide (MDP), a conserved minimal immunostimulatory motif of bacterial PGN, whereas NOD1 recognizes diaminopimelic acid-containing fragments, such as iE-DAP. Activation of these pathways triggers innate immune signaling, including NF-κB and MAPK pathways, and promotes pro-inflammatory gene expression (94–96). Thus, local intestinal dysbiosis during aging is no longer restricted to the mucosa. Instead, it may translate into a persistent systemic low-grade inflammatory stimulus. Once they reach the bone marrow via the circulation, such inflammatory and metabolic signals may contribute to reconfiguration of the bone marrow immune microenvironment.

## 4 Convergence and interaction of gut-derived signals and neuroimmune regulation within the bone marrow microenvironment

The bone marrow is a major site of bone remodeling and a critical niche for immune-cell generation, differentiation, and functional regulation. It also contributes to the maintenance of skeletal homeostasis (97). Gut microbiota dysbiosis may induce inflammatory, metabolic, and neural regulatory signals that converge within local bone marrow cellular networks. These signals may enhance osteoclastogenesis, impair osteogenesis, and contribute to bone microarchitectural deterioration (98, 99). Thus, the bone marrow can be regarded as a key convergence site through which aberrant gut-derived signals disrupt skeletal homeostasis. During aging, intestinal barrier dysfunction increases peripheral inflammatory input, while impaired autonomic regulation further weakens the fine control of inflammatory responses and bone metabolic regulation (100, 101). In parallel, bone marrow immune cells are highly sensitive to both circulating and neural cues, and alterations in their activation states can directly affect the expression of key mediators involved in bone remodeling (13, 102). Importantly, these inflammatory and autonomic pathways are unlikely to act independently. Instead, gut-derived microbial and metabolic signals may interact with altered neuroimmune regulation, thereby contributing to a bone marrow milieu that favors osteoclastogenesis and suppresses osteogenesis. **Figure 3** schematically illustrates how aged gut dysbiosis interacts with autonomic neuroimmune pathways to remodel the bone marrow neuroimmune niche.

**Figure 3:**
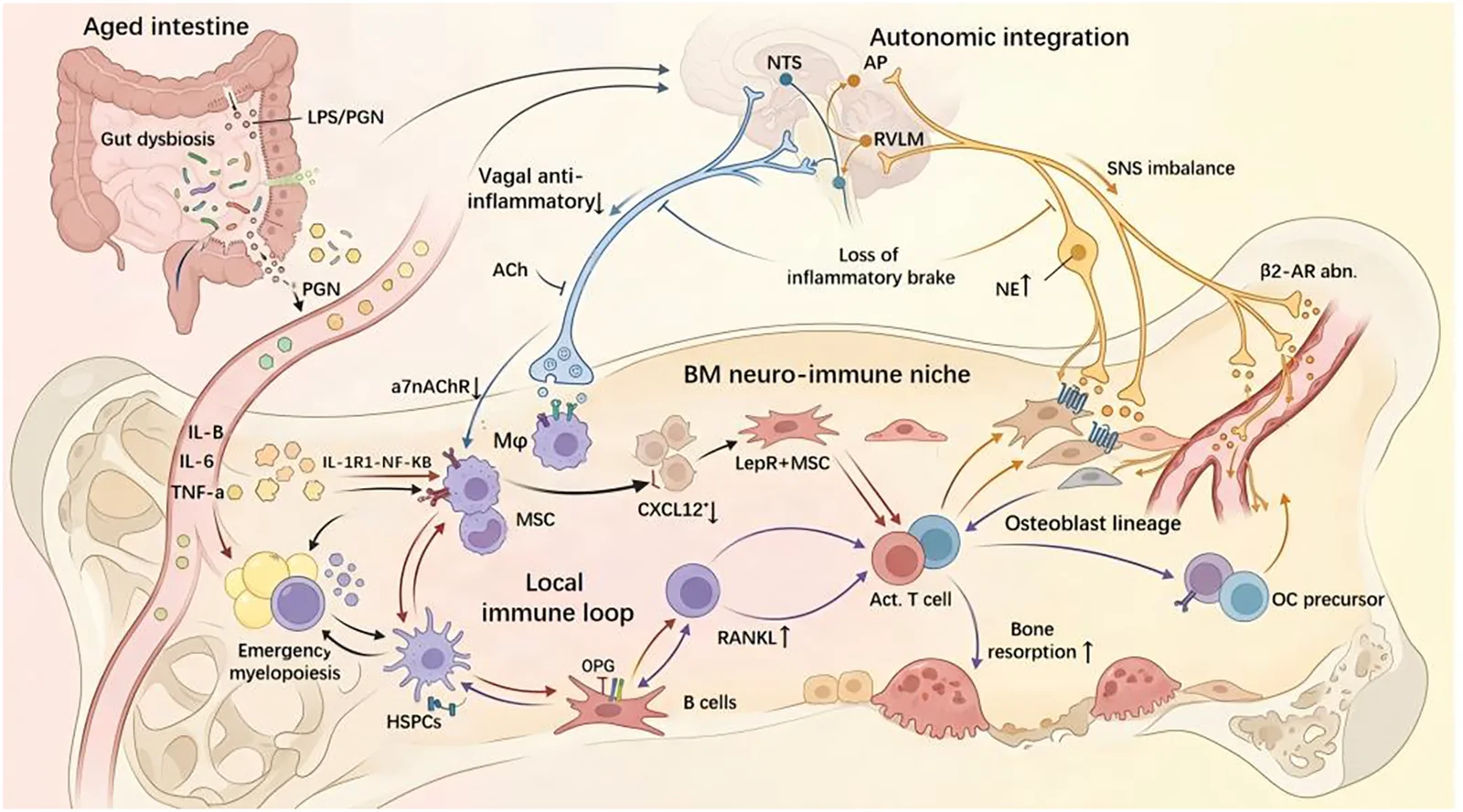
Convergent gut-derived and autonomic signals in the bone marrow neuroimmune niche. Circulating microbial products and inflammatory cytokines activate local immune and stromal-cell networks. Reduced vagal cholinergic restraint and dysregulated sympathetic norepinephrine/β2-adrenergic signaling jointly promote RANKL upregulation, OPG insufficiency, osteoclastogenesis, and bone resorption.

### 4.1 Inflammatory stimuli acting on bone marrow immune cells

Gut-derived LPS, peptidoglycan, along with other microbe-associated molecular patterns, together with inflammatory cytokines including IL-6, TNF-α, and IL-1β, can enter the circulation and deliver sustained inflammatory signals to the bone marrow microenvironment. Immune cells within the bone marrow, particularly myeloid cells, are highly responsive to persistent inflammatory cues. Gut microbiota-derived bacterial components have been reported to function as TLR4 agonists in primary macrophages derived from bone marrow, triggering TLR4-dependent activation of NF-κB and MAPK signaling, with subsequent upregulation of inflammatory genes such as Il1b (103). In parallel, elevated IL-1β can influence the bone marrow microenvironment and hematopoietic stem and progenitor cells (HSPCs) through IL-1R1-dependent inflammatory signaling. In aged bone marrow, local IL-1β drives stromal niche inflammation. This process is characterized by a reduction in endosteal osteoprogenitors, an expansion of central marrow LepR+ mesenchymal stromal cells (MSCs), sinusoidal vascular damage, and activation of emergency myelopoiesis programs within hematopoietic stem cells (HSCs) and multipotent progenitors (MPPs) (38). Chronic IL-1 signaling may therefore help sustain local inflammation in the bone marrow and promote an inflammatory amplification loop among immune and stromal cells.

An inflamed bone marrow immune microenvironment may further enhance pro-osteoclastogenic signaling. Cytokines released by macrophages and dendritic cells can promote T-cell activation. Activated T cells, in turn, increase the expression of RANKL, TNF-α and IL-17, thereby promoting osteoclastogenesis and bone resorption (104–106). Chronic inflammation may also weaken the protective counterbalance provided by B-cell-derived osteoprotegerin (OPG), shifting the RANKL–OPG balance toward osteoclastogenesis (107). Thus, peripheral inflammatory input may be converted within the bone marrow into a local pro-osteoclastogenic microenvironment through myeloid cell activation, IL-1β-driven niche inflammation and T-cell- and B-cell-mediated disruption of the RANKL–OPG axis.

### 4.2 Attenuation of the vagal anti-inflammatory pathway

The vagus nerve is a major bidirectional pathway connecting the gut and the central nervous system and plays an essential role in the autonomic regulation of immune homeostasis. The gut microbiota and its metabolites can influence vagal afferent activity through the enteric nervous system, epithelial sensing mechanisms, and immune mediator release. Following central integration, these signals may modulate descending parasympathetic output and the activity of the cholinergic anti-inflammatory pathway (108, 109). A study using Mendelian randomization and mediation analysis revealed genetic associations among gut microbiota, autonomic function, and BMD. Part of the effect of specific gut microbes on BMD was mediated by heart rate variability indices, among which pvRSA/HF and RMSSD were positively associated with BMD (16). It can be concluded that vagus parasympathetic function may be one of the important mediators but not a mere correlate linking gut microbiota dysbiosis to bone metabolic disorders.

Vagus nerve-dependent anti-inflammatory actions mediated by cholinergic signaling depend largely on acetylcholine receptors present on immune cells, specifically, the α7 nicotinic acetylcholine receptor (α7nAChR) located on macrophages, leading to inhibition of the secretion of pro-inflammatory cytokines like TNF-α, IL-1β, and IL-6 (110, 111). With advancing age, there is an impairment in parasympathetic control mechanisms; thus, the anti-inflammatory activity of cholinergic signaling via the vagus nerve may become reduced (112). Excessively high concentrations of pro-inflammatory cytokines in bone tissue result in the promotion of RANKL-related osteoclastogenic pathways, increased osteoclastogenesis, bone resorption, as well as osteoblast dysfunctions (113). Beyond its indirect anti-inflammatory effects, cholinergic neural signaling may also exert direct regulatory effects on bone. Cholinergic nerve fibers have been identified in the periosteum (114), and osteogenic lineage cells express cholinergic receptors and related signaling molecules (115), indicating that local bone tissue possesses the molecular machinery required to respond to acetylcholine signaling. Moreover, there are reports demonstrating that acetylcholine-stimulation could modulate osteoblasts activity by regulating their proliferation, differentiation, and mineralization (116). In development studies, exogenous acetylcholine application or choline acetyltransferase-dependent procedures were shown to stimulate bone mineralization (117).

### 4.3 Abnormal sympathetic output and imbalance in bone remodeling

Whereas the vagus nerve mainly contributes to anti-inflammatory homeostasis, the sympathetic nervous system more directly influences the local adrenergic microenvironment of bone. Sympathetic nerve fibers are found throughout the periosteum, bone marrow, and perivascular regions of bone tissue (118, 119). Norepinephrine released from these fibers can regulate intraosseous vascular homeostasis, osteogenesis-associated angiogenesis, and bone mass maintenance via adrenergic receptor β2-AR-mediated signaling (120). Histological studies have shown that both sensory and sympathetic nerve fibers are detectable in the periosteum, cortical bone, and bone marrow of the mouse femur, with the highest density observed in the periosteum. With aging, the cambium layer of the periosteum becomes markedly thinner, while the total numbers of sensory and sympathetic nerve fibers decrease. In aged mice, sympathetic nerve fiber density in cortical bone is decreased by approximately 75% compared with adult mice (121). These findings indicate that aging affects not only bone metabolic cells but also the local neural network architecture of bone.

Aging is also associated with systemic abnormalities in sympathetic regulation. Studies of the human autonomic nervous system have shown increased central sympathetic activity but reduced peripheral sympathetic transduction in older adults. This pattern suggests an uncoupling between sympathetic nerve firing and peripheral effector responses (122). Thus, the adrenergic influence on bones can depend not only on the local density of sympathetic fibers in bones, but also on central sympathetic output and local neurotransmitter release capability. Some recent research also demonstrates that the central-peripheral sympathetic regulation axis does not work in isolation, but is constantly modulated by the gut signals. The gut microbiota and its metabolites may signal to autonomic integration centers through visceral afferent pathways, particularly the vagus nerve. These centers include the nucleus tractus solitarius (NTS), dorsal motor nucleus of the vagus (DMV), area postrema (AP), and lateral paragigantocellular nucleus/rostral ventrolateral medulla (LPGi/RVLM). These signals may subsequently influence peripheral sympathetic output (123). In the context of aging, this regulatory effect may be more readily amplified. Enhanced central sympathetic drive and impaired peripheral effector transmission coexist during aging; thus, even when local bone innervation is reduced, bone tissue may remain exposed to abnormal systemic sympathetic signaling. Animal studies further support the notion that overactivation of central stress-related noradrenergic circuits can affect bone through skeletal sympathetic outflow (120).

In addition to centrally integrated regulation, a dynamic feedback loop exists within the intestinal wall between noradrenergic signaling and microbial metabolism. Activation of intestinal sympathetic nerves can increase local norepinephrine release and regulate epithelial cell function, mucosal barrier homeostasis, and the exchange of luminal and host-derived substances through adrenergic receptors (124, 125). SCFAs can regulate peripheral sympathetic activity at the level of the sympathetic ganglia via GPR41/FFAR3 and promote norepinephrine (NE) release from sympathetic neurons (126, 127). Although these pathways operate at different regulatory levels, they may converge to produce dysregulated norepinephrine tone and amplified adrenergic signaling in bone. Aberrant norepinephrine signaling can inhibit bone formation through β2-ARs expressed on osteoblasts and osteoprogenitor cells, while promoting RANKL expression and osteoclast-mediated bone resorption (128, 129). Moreover, dysregulated sympathetic adrenergic signaling may impair intraosseous type H vessels and disrupt angiogenesis–osteogenesis coupling, thereby further aggravating the imbalance in bone remodeling (120).

### 4.4 Intestinal inflammation, vagal signaling, and sympathetic activation as a coordinated amplification network

Inflammatory input associated with gut dysbiosis can disrupt autonomic nervous function. Impaired intestinal barrier integrity promotes the translocation of gut-derived microbial products, such as LPS, and induces the release of local inflammatory mediators. These inflammatory signals can be encoded by vagal sensory afferents and then integrated into autonomic regulation. Vagal afferents and nodose ganglion neurons express TLR4, IL-1 receptors, and TNF receptors, allowing them to directly sense LPS and pro-inflammatory cytokines. Persistent inflammatory stimulation may lead to abnormal neuronal excitability, disturbed calcium homeostasis, and altered thresholds for inflammatory sensing (92, 130). Inflammatory signals may also remodel sympathetic neural regulation. In sympathetic ganglia, LPS can induce reactive changes in satellite glial cells and interfere with Orai1/STIM1-mediated calcium influx. These findings suggest that inflammation disrupts the neuron-glia microenvironment required for stable sympathetic output (131). Under chronic inflammatory conditions, peripheral sympathetic fibers may undergo structural remodeling. In a CFA-induced model of inflammatory arthritis, TH^+^ sympathetic nerve fibers show ectopic sprouting, increased density, disorganized morphology, and neuroma-like structures in the synovium and femoral periosteum (132).

Abnormal sympathetic output can reshape the bone marrow myeloid microenvironment through norepinephrine and increase the inflammatory responsiveness of the monocyte-macrophage lineage. During sustained sympathetic activation, norepinephrine released from sympathetic nerve terminals and catecholamine-producing cells acts on granulocyte-macrophage progenitors expressing β2-adrenergic receptors. This promotes progenitor proliferation and myeloid differentiation, thereby expanding the hematopoietic supply of monocytes and macrophages (133). Catecholamines can also induce trained immunity-like reprogramming in monocytes, leading to higher TNF-α and IL-6 production after subsequent LPS restimulation. This effect is associated with β-adrenergic receptors and cAMP/PKA signaling (134).

Thus, autonomic dysfunction may serve as an important bridge between gut-derived inflammatory input and bone marrow myeloid inflammation (135). Reduced vagal anti-inflammatory restraint weakens cholinergic feedback and allows cytokine release from monocytes and macrophages to persist (136). In parallel, enhanced sympathetic outflow reshapes the neural regulatory environment of the bone marrow, increases hematopoietic activity, promotes myeloid cell mobilization, and heightens inflammatory recall responses (9). Together, these signals may shift the bone marrow from a passive target of peripheral inflammation to an amplification node that sustains inflammatory responses. This may form a pathological loop linking gut inflammation, autonomic imbalance, and bone marrow myeloid inflammation.

## 5 Therapeutic implications of age-related gut–neuroimmune dysregulation

The development and progression of age-related osteoporosis involve a series of interconnected yet potentially modifiable pathological processes, ranging from gut microbiota dysbiosis to remodeling of the neuroimmune microenvironment in the bone marrow (137). Unlike conventional strategies that mainly focus on calcium supplementation, inhibition of bone resorption, or stimulation of bone formation, interventions targeting the gut–neuroimmune–bone axis may provide multilayered regulation. Such an approach will not only account for the high degree of response variance to traditional treatments among elderly patients, but also provide new ways for stratifying risk and predicting early onset and personalized intervention. However, most of these methods still exist mostly in the form of research conducted in animals or early-stage clinical investigations into mechanisms. The sustainability of their therapeutic effects is yet to be validated (138). **Figure 4** schematically illustrates potential biomarkers and interventions targeting gut dysbiosis, neuroimmune imbalance, and bone marrow inflammation remodeling.

**Figure 4:**
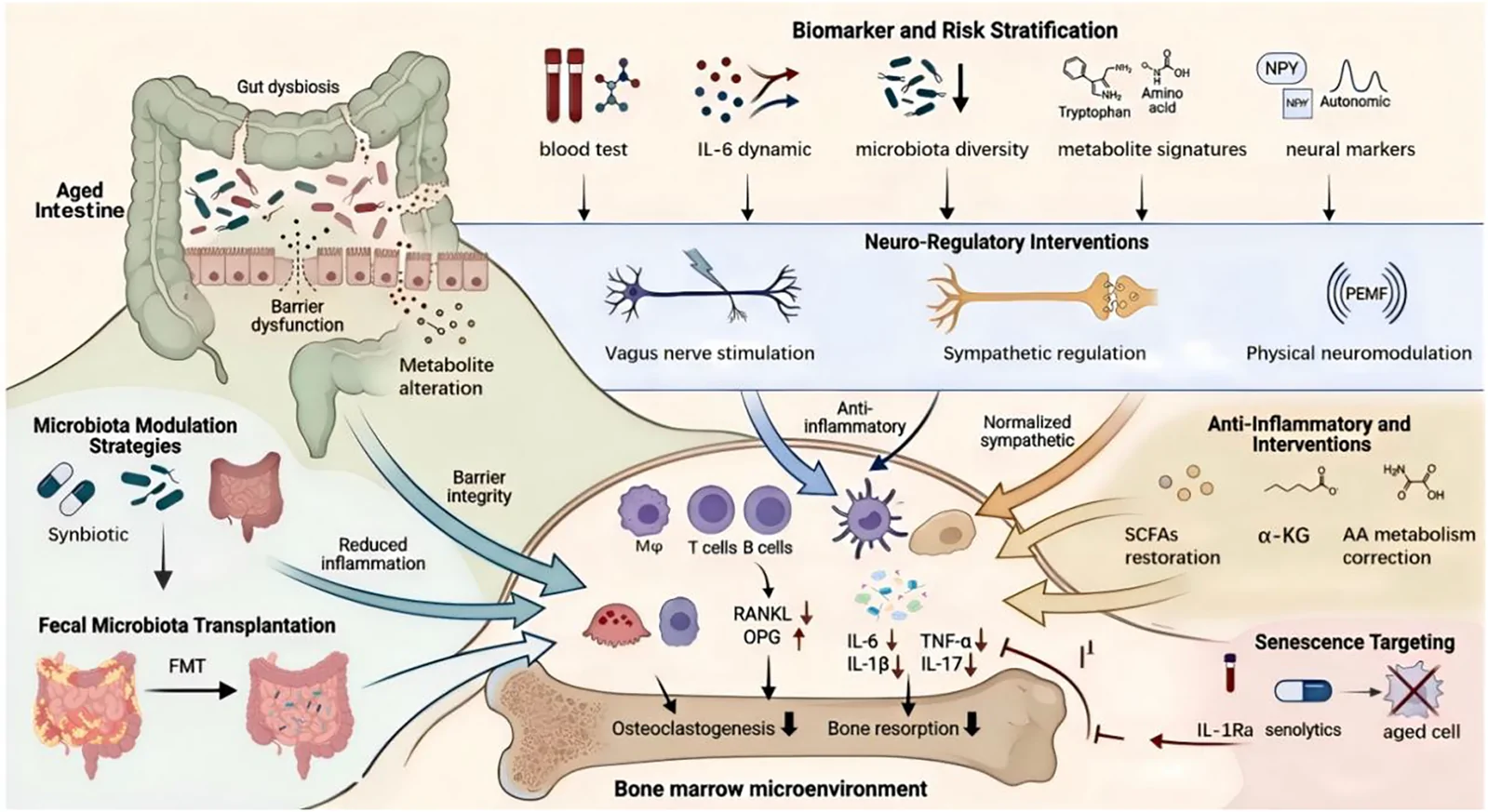
Biomarker-guided and therapeutic strategies for the gut–neuroimmune–bone axis. Candidate biomarkers include inflammatory cytokines, gut microbial profiles, microbial metabolic fingerprints, and neural indicators. Potential interventions targeting the gut microbiota, neuroimmune pathways, bone marrow inflammation, metabolism, and cellular senescence may help reduce osteoclastogenesis and bone resorption.

### 5.1 Biomarkers for risk stratification

The importance of biomarkers for age-related osteoporosis goes beyond the ability to classify already existing bone loss. It includes the possibility of identifying patients at higher risk of developing this condition, predicting the progression of the disease, and stratifying patients. While bone mass loss can occur without any symptoms, changes in BMD and structure tend to take a long time to evolve. Imaging indicators may not be sufficient to detect the early functional abnormalities. Imaging-based indicators alone may therefore fail to capture early functional abnormalities (139). Biomarkers reflecting inflammatory activation, impaired neural regulation, or gut microbiota dysbiosis before overt skeletal damage may provide earlier warning signals and help identify patients who are more likely to benefit from specific interventions.

Among inflammation-related markers, longitudinal changes may offer greater predictive value than single measurements. Longitudinal studies have shown that temporal changes in serum IL-6 levels significantly predict loss of BMD at the whole-body, hip, and spinal sites and are associated with elevated bone resorption (140). These findings indicate that inflammatory cytokines are not simply secondary consequences of bone loss, but may reflect an early and persistent imbalance in bone remodeling. Compared with a single marker, combined abnormalities in multiple inflammatory indicators may better capture the overall burden of chronic low-grade inflammation, and may therefore be better suited for identifying individuals with “inflammaging-driven” bone loss (141).

Neuroregulatory molecules may provide an additional layer of information distinct from inflammatory markers. Increased expression of neuropeptide Y (NPY) and its Y1 receptor (Y1R) has been detected in bone tissue from older patients with osteoporosis. Their expression is correlated with bone volume fraction, expressed as bone volume/tissue volume (BV/TV) (142), suggesting that dysregulation of the neuropeptide axis is not only associated with established microarchitectural damage, but may also reflect a persistent shift in local skeletal regulatory networks. Compared with inflammatory markers, these molecules may more specifically indicate a “neuroimmune dysregulation–associated” pattern of bone loss, thereby supporting future mechanistic subtyping and individualized intervention strategies.

Gut microbiota and derived metabolites may serve as upstream indicators of age-related osteoporosis risk. Studies have shown that individuals with low bone mass exhibit reduced overall gut microbial richness, along with lower relative abundances of Roseburia, Bifidobacterium, and Lactobacillus, which are positively correlated with BMD and T-scores (5). Multi-omics analyses further indicate that osteoporosis-related microbial signatures are not limited to taxonomic differences, but are closely associated with alterations in tyrosine and tryptophan metabolism, as well as degradation of branched-chain amino acids (143). Metagenomic studies in middle-aged and older adults have shown that functional pathways related to butyrate production and L-arginine biosynthesis are positively associated with BMD. These associations remained significant after adjustment for age, diet, and lifestyle-related factors (144). Thus, compared with the abundance of individual bacterial taxa, microbial functional features and metabolite profiles may more directly reflect disease mechanisms and may serve as more informative candidate markers for future risk prediction and population stratification.

### 5.2 Modulation of the gut microbiota

#### 5.2.1 Probiotics and prebiotics

Probiotics and prebiotics represent some of the most widely investigated microbiota-targeted interventions. In a randomized, placebo-controlled, double-blind trial, 12-month supplementation with Lactobacillus reuteri ATCC PTA 6475 significantly attenuated the decline in total tibial volumetric BMD among women aged 75–80 years with low bone mass (78). Mechanistically, the potential benefits of these interventions may extend beyond changes in microbial taxonomic composition. They may support intestinal barrier integrity, alleviate chronic low-grade inflammation, and suppress osteoclast differentiation and bone resorption via metabolites such as short-chain fatty acids (65, 145, 146). These interventions may therefore serve as long-term adjunctive strategies that target upstream pathological drivers of bone loss.

The bone-protective effects of probiotics and prebiotics may depend primarily on their ability to reshape the metabolic functions of the gut microbiota, rather than on alterations in the relative abundance of individual bacterial taxa (147). SCFAs and tryptophan metabolites may therefore serve as key functional indicators of intervention efficacy, reflecting dietary fiber fermentation capacity and the balance of host-microbiota tryptophan metabolism, respectively. SCFAs contribute to intestinal barrier homeostasis and may attenuate the impact of chronic low-grade inflammation on the bone marrow immune microenvironment. Tryptophan metabolites may further alleviate inflammation-related bone resorption by increasing beneficial indole derivatives and limiting excessive diversion toward the kynurenine pathway (148).

However, their clinical application still faces substantial limitations. Baseline microbiota composition, dietary patterns, metabolic status, and medication exposure vary considerably among individuals. Therefore, the impact of the same bacterial strain or prebiotic on different populations might not be consistent. Furthermore, the choice of strain, the efficiency of colonization, optimal dosage, long term safety and the effect of combination with current anti-osteoporotic drugs are not supported by consistent human evidence. It is therefore necessary to go beyond the assumption of universal efficacy in the future development. It would be more rational to combine microbial subtyping and metabolic profiling to choose beneficial strains or combination regimens for improving precision and reproducibility.

#### 5.2.2 Fecal microbiota transplantation

Fecal microbiota transplantation (FMT) constitutes another microbiota-based strategy that more closely resembles whole-community reconstruction. Studies have shown that transferring microbiota from young donors to aged recipients can enhance intestinal barrier integrity and immune homeostasis (149). By contrast, transplanting microbiota from osteoporotic subjects to recipient animals resulted in development of osteoporosis phenotypes, manifested by increased bone loss and barrier dysfunction and inflammatory activation (150). The results imply that it is possible for microbial dysbiosis to cause bone loss pathologically. In addition, such a strategy could theoretically target the three processes of barrier function, immune regulation, and bone metabolism at the same time. However, there is still a lack of convincing data regarding its safety, donor qualification, sustained effects, and effectiveness in age-related osteoporosis. Currently, it is better to consider FMT as a tool for research rather than clinical application.

### 5.3 Modulation of neuroimmune pathways

In older adults, minimally invasive and repeatable physical or neuromodulatory interventions may offer practical advantages, particularly for individuals with poor drug tolerance, chronic inflammatory conditions, or functional decline. Compared with strategies that directly target the microbiota, neuromodulation acts at an intermediate level, where gut microbiota dysbiosis may be translated into abnormalities in the bone marrow microenvironment.

Vagus nerve-associated interventions are intended to restore the cholinergic anti-inflammatory brake, inhibit the activation of pro-inflammatory immune cells, including macrophages, and limit the transmission of inflammatory mediators, such as TNF-α, IL-1β, and IL-6, to the bone marrow microenvironment. Animal studies indicate that targeted vagus nerve stimulation(VNS) may alleviate osteoporotic phenotypes, suggesting potential translational value (151). Mechanistically, the major value of this approach may lie in its use as a research tool to determine whether the cholinergic anti-inflammatory pathway contributes to age-related osteoporosis. Human evidence directly evaluating VNS for age-related osteoporosis remains very limited. A small cohort study in patients with epilepsy suggested that lumbar spine BMD may increase after VNS implantation (152). In rheumatoid arthritis, studies of implantable VNS have provided proof-of-concept evidence that the cholinergic anti-inflammatory pathway can be modulated in human chronic inflammation and inflammation-related bone destruction (153–155). By contrast, studies of non-invasive auricular VNS have yielded inconsistent results. This suggests that stimulation site, stimulation parameters, and study design may substantially influence therapeutic efficacy (156, 157).

Sympathetic nervous system modulation may have additional translational significance. Sympathetic dysregulation can directly affect local NE/β2-AR signaling, RANKL expression, and type H vessel homeostasis in bone (120, 158). Thus, studies targeting sympathetic tone or downstream adrenergic signaling may help determine whether bone tissue is chronically exposed to an abnormal pro-inflammatory and pro-resorptive neural milieu. In the future, combined assessment of neural functional indicators, immune cell activation, and bone turnover markers may help identify patients whose bone loss is predominantly driven by neuroimmune mechanisms.

Beyond classical neurostimulation, some non-invasive physical therapies may also act through neural pathways. Pulsed electromagnetic fields (PEMFs) have been reported to increase femoral trabecular bone mass, improve trabecular microarchitectural structure, and promote bone formation in aged male mice. These osteogenic effects are markedly weakened after sensory nerve depletion or loss of sensory nerve-derived Sema3A, indicating that the effect is at least partly dependent on sensory nerve-related pathways (37). Although these interventions should not be considered equivalent to targeted vagal or sympathetic modulation, they suggest that neuromodulatory strategies for age-related osteoporosis may extend beyond electrical or pharmacological approaches to a broader field of nerve-related physical therapies.

### 5.4 Targeting bone marrow inflammation and bone remodeling

Interventions targeting bone marrow inflammation may focus on suppressing inflammatory niches, blocking osteoclastic effector mechanisms, and restoring osteogenic function. The IL-1β–IL-1R1 axis is currently one of the more clearly defined therapeutic targets. Studies in aged mice have shown that local IL-1 signaling in the bone marrow promotes stromal niche inflammation and hematopoietic decline. Treatment with the IL-1 receptor antagonist anakinra partially ameliorates degeneration of the bone marrow niche, dysfunction of hematopoietic stem cells, and impaired regenerative capacity. These findings suggest that anti-IL-1 therapy may help lower the inflammatory burden within the aging bone marrow (38).

In the context of osteoclast-driven bone resorption, senescent cell clearance is particularly consistent with the pathophysiology of age-related osteoporosis. Farr et al. found that, in 20–22-month-old mice, genetic elimination of p16-positive senescent cells or 2–4 months of treatment with senolytic agents or JAK inhibitors increased bone mass and strength while improving bone microarchitecture. These skeletal protective effects were mainly characterized by reduced bone resorption, while bone formation was maintained or enhanced (159).

For impaired bone formation, metabolic intervention provides another therapeutic avenue. Supplementation with α-ketoglutarate (α-KG) enhances bone mass and improves bone microarchitecture in aged mice. *In vitro* studies further show that αKG improves the phenotype of aged mesenchymal stem cells through modulation of epigenetic status and enhancing their osteogenic differentiation capacity (160). These findings suggest that metabolic regulation targeting MSC senescence and impaired osteogenic potential may help restore bone formation in age-related osteoporosis.

## 6 Summary and outlook

Age-related osteoporosis represents a systems-level disorder of the gut–neuroimmune–bone marrow axis. Its progression reflects the convergence of microbial, inflammatory, and autonomic disturbances within the marrow niche, shifting the disease paradigm from local bone-cell dysfunction to multisystem regulatory failure.

### 6.1 Limitations of current evidence

Current research still faces major translational limitations. Causal evidence linking the gut microbiota, immune inflammation, and bone remodeling is mainly derived from animal models. These include ovariectomy models, D-galactose-induced aging models, antibiotic depletion models, and FMT models. These models are useful for dissecting specific mechanisms. However, they cannot fully reproduce the long-term interactions among diet, medication exposure, comorbidities, hormonal changes, and lifestyle factors during human aging. Available human trials suggest that the skeletal effects of microbiota-based interventions are highly dependent on the strain, study population, and clinical endpoint. Jansson et al. found that a mixture of three Lactobacillus strains slowed lumbar spine BMD loss in early postmenopausal women. However, this effect was not consistently observed at all skeletal sites (161). Takimoto et al. reported that Bacillus subtilis C-3102 improved some BMD parameters and affected bone resorption markers in healthy Japanese postmenopausal women. This suggests that strain characteristics and regional population background may influence treatment responses (162). In the multicenter, randomized, double-blind, placebo-controlled SBD111 synbiotic trial, Schott et al. found no significant reduction in bone loss in the overall cohort of early postmenopausal women. Potential benefits were observed only in subgroups with osteopenia or higher BMI (163). The gut microbiota is strongly influenced by ethnicity, geography, diet, and lifestyle. Therefore, microbiota-bone associations derived from a single region or ethnic group should not be directly generalized to other populations (164, 165).

FMT studies are also limited by donor heterogeneity, colonization stability, and long-term safety concerns. The concept of “super donors” suggests that efficacy may depend on donor microbial diversity, key species, and donor-recipient compatibility, rather than simple microbiota replacement (166). Older adults and immunocompromised individuals are more vulnerable to infection and metabolic disturbances. Therefore, FMT requires strict pathogen screening, standardized preparation procedures, and long-term follow-up (167).

Neuromodulatory interventions also face translational and clinical implementation challenges. Unlike pharmacological agents or microbiota-based products, neuromodulation is a bioelectronic intervention that depends on the device, target site and stimulation parameters. Its efficacy may be influenced by the stimulation site, electrode placement and parameter settings (167, 168). Its safety and scalability also require standardized procedures and systematic adverse-event monitoring (169).

### 6.2 Future perspectives

Future research should move beyond the validation of single mechanisms and toward multilayered, translationally oriented integrative studies. In basic research, it will be important to clarify the dynamic causal relationships among gut microbiota-derived metabolites, neural activity, inflammatory mediators, and bone marrow cell states. In population studies, longitudinal cohorts should be combined with multi-omics analyses, autonomic function assessment, bone turnover markers, BMD measurement, and evaluation of bone microarchitecture. This approach may help identify reproducible microbial signatures and neuroimmune phenotypes.

For clinical interventions, multicenter randomized controlled trials with long-term follow-up are needed. These trials should systematically assess the efficacy, durability, and safety of probiotics, prebiotics, FMT, neuromodulation, and anti-inflammatory strategies. A mechanism-guided stratification framework should integrate gut microbial profiles, neuroimmune status, and bone marrow inflammatory features. Such a framework could support early identification, risk prediction, and precision intervention in age-related osteoporosis.
